# Xpert MTB/RIF assay for diagnosis of pulmonary tuberculosis in sputum specimens in remote health care facility

**DOI:** 10.1186/s12866-015-0566-6

**Published:** 2015-10-19

**Authors:** Dereje Assefa Geleta, Yoseph Cherinet Megerssa, Adugna Negussie Gudeta, Gizachew Taddesse Akalu, Melaku Tesfaye Debele, Kassu Desta Tulu

**Affiliations:** International Organization for Migration, P.o.box- 25283, Addis Ababa, code 1000 Ethiopia; College of Veterinary Medicine and Agriculture Addis Ababa University, Addis Ababa, Ethiopia; Jigjiga Universities, Jigjiga, Ethiopia; Ethiopian Medical Laboratory Association, Addis Ababa, Ethiopia; International Clinical Laboratories, Addis Ababa, Ethiopia; College of health sciences Addis Ababa University, Addis Ababa, Ethiopia

**Keywords:** NPV, PPV, Sensitivity, Specificity, Xpert MTB/RIF assay, Remote health facility

## Abstract

**Background:**

Xpert MTB/RIF assay is considered as a great advance over conventional smear and culture in the diagnosis of TB and MDR-TB by simultaneously detecting *M.tuberculosis* and rifampicin resistance bacilli. However, very little information regarding the performance characteristics of Xpert MTB/RIF assay is available in Ethiopia. Therefore, the purpose of this study was to evaluate the performance of Xpert MTB/RIF assay compared to conventional sputum smear and culture methods for the diagnosis of pulmonary tuberculosis in remote health care facility.

**Methods:**

A paired expectorated sputum samples were obtained from 227 consecutively recruited patients with signs and symptoms suggestive of tuberculosis at Karamara hospital during December 2013 to May 2014. One of the sputum specimen was tested directly by Ziehl-Neelsen staining and Xpert MTB/RIF assay without NALC-NaOH decontamination. The other of pair of sputa specimen was cultured for isolation of TB bacilli by conventional methods. Diagnostic performance of Xpert MTB/RIF assay and AFB smear microscopy were calculated against culture as the gold standard.

**Results:**

Overall 25.5 % (58/227) samples were positive for *Mycobacterium tuberculosis* complex (MTBC) by MGIT and/or LJ media of which 36.2 % (21/58) and 65.5 % (35/58) were positive by AFB smear microscopy and Xpert MTB/RIF respectively. The sensitivity, specificity, as well as the positive and negative predictive value of Xpert MTB/RIF assay were 65.5 % (95 % CI: 53.3–77.7 %), 96.3 % (95 % CI: 93.4–99.2 %), 86.4 % (95 % CI: 76.2–96.5 %), and 88.6 % (95 % CI: 83.9–93.3 %) respectively. Eighteen of 58 (31 %) cases that were smear microscopy negative, were positive by Xpert MTB/RIF assay.

**Conclusions:**

Although Xpert MTB/RIF assay demonstrated high sensitivity in detecting MTBC in sputum specimens compared with conventional AFB smear microscopy, it demonstrated suboptimal sensitivity in smear negative patients compared to conventional culture.

## Background

Tuberculosis (TB), which is caused by *Mycobacterium tuberculosis* (MTB) complex, is a major global health problem infecting millions of people every year, with a particular heavier burden in the developing world. Ethiopia is one of the 22 highest TB burden countries. The national population-based TB prevalence survey conducted in 2010/11 revealed that the prevalence of smear positive TB among adults and all age groups was found to be 108 and 63 per 100,000 respectively. The prevalence of bacteriological confirmed TB was found to be 156 per100, 000 and the prevalence of all forms of TB in Ethiopia is estimated to be 240 per 100, 000. There were an estimated 15,000 deaths (18 per 100,000 populations) directly related to TB infection, excluding co-morbidity of HIV related deaths [1–3].

Diagnosing and detecting active TB and multi-drug resistant (MDR) strains are essential to interrupt transmission of the disease in the community [4]. Isolation of the bacteria using conventional solid culture and drug susceptibility testing (DST) is the gold standard. Liquid culture and molecular line probe assays were also introduce for rapid detection of MDR-TB. However these methods require long turnaround time, expensive laboratory infrastructure, extensive bio-safety precaution, and specialized laboratory personnel seldom found in primary health care facilities in developing countries [5].

Xpert MTB/RIF assay is a new rapid molecular test that asserted that it was able to overcome many of the current operational difficulties in TB diagnosis. WHO endorsed Xpert MTB/RIF assay in 2010 for use in TB prevalent resource limited countries as a first line diagnostic test for rapid diagnosis of TB in HIV infected patients or for management of MDR-TB suspect [6].

Ethiopia is among one of the 21 recipient countries to implement the TBXpert project, however, performance related data from high suspect of TB patients at the point of treatment settings in Ethiopia is scarce. There is little data to inform recommendations to use the assay for testing sputum samples when investigating suspected pulmonary TB. Therefore the present study is aimed to give information on the performance of Xpert MTB/RIF assay on patients with suspected of having pulmonary TB from sputum samples and its agreement with the current TB diagnostic test in the country.

## Methods

### Settings and study design

A cross-sectional study was conducted on patients with suspect pulmonary TB to evaluate the performance of Xpert MTB/RIF assay for detection of MTBC. The study was conducted at Karamara hospital in Jigjiga town. Jigjiga is the capital of Somali region which is located 631 km away from Addis Ababa, the capital city of Ethiopia. The town is located in the eastern part of Ethiopia and 60 km west of the border with Somalia.

### Study participants

Patients that were the age of 18 and above were eligible if they presented with signs and symptoms suggestive of tuberculosis and/or who have a suggestive TB chest x-ray and had a prior history of tuberculosis or suspected MDR TB. Patients who were taking TB treatment for more than 2 weeks prior to the initiation of the study were excluded. Using consecutive sampling technique a total of 227 patients were enrolled in the study. The study was conducted from December 2013 to May 2014.

### Specimen collection

Two consecutive expectorated morning sputum samples were collected on the same day from the enrolled patient. The minimum acceptable volume of sputum was 2 ml in each collection tube. One of the sputum specimen was tested directly by Ziehl-Neelsen staining microscopic examination and Xpert MTB/RIF assay. The other of pair of sputum spacemen was cultured for isolation of TB bacilli by conventional methods. Specimens for TB culture were stored at appropriate temperature after collection. The sputa were placed on an ice packs in a cool box and transported to International Clinical Laboratories (ICL) TB laboratory within three days of collection and analyzed.

### Laboratory methods

Ziehl-Neelsen direct AFB smear, Xpert MTB/RIF assay, Solid (LJ) and liquid (MGIT 960) medium for TB culture, were included. Xpert assay and AFB smear was performed at Karamara hospital from one fresh sputum sample and on the other TB culture was performed at ICL. Smear grading was performed according to the WHO/International Union Against Tuberculosis and Lung Disease method [7].

### Xpert MTB/RIF assay

Sputum samples are treated with sample reagent (SR) containing NaOH and isopropanol. The SR is added using a 2 to 1 ratio of the sputum sample, homogenized and incubated for 15 min at room temperature. The treated sample is transferred into the cartridge, the cartridge is loaded into the GeneXpert instrument, and an automatic process completes the remaining assay steps [8].

### Mycobacterial culture

The sputa were collected in a sterile container which was placed on an ice pack in a cool box, sent to ICL and processed within the same day of arrival. Digestion-decontamination of sputum was performed by N-acetyl L-cysteine (NALC) Sodium Hydroxide method. Both LJ and MIGT 960 culture media were inoculated and incubated for 6 weeks on MGIT and 8 weeks on LJ culture. A culture was considered positive if MTB growth was confirmed on either LJ or MGIT media. A culture was considered negative if no growth was confirmed on both LJ and MGIT media or if one culture result was negative and the other is contaminated. A culture was considered contaminated if both LJ and MGIT demonstrated contamination. The positive culture was confirmed to contain *M.tuberculosis* complex by using Capilia Tb antigen test.

### Quality assurance and data collection

Data collection was conducted after the participant was informed the purpose of the study and when gave consent. Demographic data was collected from the TB registration book. Sputum sample and data collection was conducted by trained laboratory personnel. The sputa were collected in a sterile container (falcon tube) which was placed on an ice pack in a cool box during sample transportation to the laboratory. All laboratory tests were performed by well-trained laboratory personnel. Standard operational procedures of the host laboratory were used to ensure the reliability and validity of test result. To avoid subjective interpretation of test results the laboratory personnel processing the sputum samples for TB culture and Xpert MTB/RIF were blinded to the results of the other test.

### Statistical analysis

The results of Xpert assay was compared with smear microscope and culture for the presence of AFB and MTBC respectively. The data collected from TB registration book were entered into an excel spreadsheet and transmitted and analyzed by SPSS version-20.0. The sensitivity, specificity, positive and negative predictive values for Xpert MTB/RIF assay and AFB smear were calculated using TB culture as the gold standard. The sensitivity and specificity between Xpert MTB/RIF assay and AFB smear were determined using McNemar’s test. The association between test statuses and independent variables were compared using Fisher’s exact test and Wilcoxons’s rank sum test for categorical and continuous variables respectively. Confidence intervals (CI) were calculated by exact method. Two-sided *P-*values less than 0.05 was considered as significant.

### Ethical considerations

Ethical clearance (Protocol number DRERC062/13/MLS) was collected from research and ethics review committee of the department of medical laboratory sciences, school of allied health sciences at Addis Ababa University. The participants were given the opportunity reading the consent form, and when agree upon was involved in the study. Confidentiality was kept throughout the study. The laboratory findings were provided to the study participants and those in need of medical attention were referred to their treating physicians.

## Results

### Demographic and diagnostic characteristics

In the current study, 227 paired sputum specimens ordered for routine AFB smear microscopy in the TB clinic of Karamara hospital were included. Among the sputum specimens, 63.0 % (143/227) and 37.0 % (84/227) were obtained from male and female study participants respectively resulting male to female ration of 1.7 to 1. The age of the study participants ranged from 18 to 82 years with median age of 35 years (Table 1).

**Table 1:** Diagnostic characteristics of study population by sex at Karamara hospital, Jigjiga, Ethiopia (*n* = 227)

Microbiological classification						
Sex	Number (n)	Percent (%)	Smear (+) Culture (+)	Smear (−) Culture (+)	Culture (−)	Contaminated samples
Male	143	63.00 %	14(6.2 %)	23(10.1 %)	104(45.8 %)	2(0.8 %)
Female	84	37.00 %	7(3.1 %)	14(6.6 %)	62(27.3 %)	1(0.4 %)
Total	227	100 %	21(9.3 %)	37(16.3 %)	166(73.1 %)	3(1.3 %)

A pair of spontaneously expectorated morning sputa specimen were concurrently collected from each patient on the same day. One arbitrarily selected sample was submitted for MGIT 960 and LJ inoculation. The second fresh sputum sample was analyzed for Xpert MTB/RIF and direct AFB smear. A confirmed positive culture of MTBC was used as a reference standard. All 227 study participants had cultures (both LJ and MGIT), AFB smear, and Xpert MTB/RIF tests. Since culture was used as a reference for the study, results with contaminated cultures for both LJ and MGIT were excluded (Table 1).

Overall, 224 sputa had an interpretable result for MGIT and/or LJ. Three sputa with a MGIT and LJ final result of “contaminated” were excluded from this analysis. Out of 224 specimens, 58 (25.9 %) were positive for MTBC by MGIT and/or LJ media. When the specimen stratified by AFB smear microscopy status; 9.3 % (21/227) had smear-positive, culture-positive TB, whereas 16.3 % (37/227) had smear-negative, culture-positive TB (Table 1). The median time for *M.tuberculosis* liquid culture positivity was 15 days (range 4–27 days), 10 days for smear positive and 19 days for smear-negative cases (*P <* 0.05). Compared with culture, the sensitivity, specificity, PPV, and NPV for AFB smear microscopy examination was 36.2 % (95 % CI: 23.8 %–48.6 %), 99.4 % (95 % CI: 98.2–100 %), 95.5 % (95 % CI: 86.8–100 %), and 80.4 % (95 % CI: 74.5–855 %) respectively.

### Performance of Xpert MTB/RIF assay

A total of 227 sputum samples were run in Xpert MTB/RIF assay. Out of 227, 98.2 % (223/227) samples gave an interpretable Xpert MTB/RIF assay result. Four samples (1.8 %) with an Xpert final result of “Invalid” were excluded from the analysis. From the valid Xpert MTB/RIF results; 19.4 % (44/227) were positive and 78.8 % (179/227) were negative. Twenty MTBC (8.8 %) isolated from culture were not detected by Xpert MTB/RIF assay. Six false positive (2.6 %) Xpert MTB/RIF results were recorded in the study (Table 2).

**Table 2:** Xpert MTB/RIF assay and culture results

Culture result				
Xpert MTB/RIF	MTB	Negative	Contaminated	Total
Positive	38 (16.7 %)	6 (2.6 %)	0	44 (19.4 %)
Negative	20 (8.8 %)	156 (68.7 %)	3 (1.3 %)	179 (78.9)
Invalid	1 (0.4 %)	3 (1.3 %)	0	4 (1.8 %)
Total	59 (25.9 %)	165 (72.6 %)	3 (1.3 %)	227 (100 %)

Eighteen (7.9 %) AFB smear negative samples were Xpert MTB/RIF and culture positive. When the specimens stratified by AFB smear status, Xpert MTB/RIF assay has a sensitivity of 95.2 % (95 % CI: 86.1–100 %) for smear-positive-culture positive specimens and a sensitivity of 48.6 % (95 % CI: 32.5–64.8 %) for smear-negative-culture positive specimens. The sensitivity was higher for smear-positive than for smear-negative specimens (*p* = 0.007). Overall, the sensitivity of Xpert MTB/RIF was 65.5 % (95 % CI: 53.3–77.7 %), specificity was 96.3 % (95 % CI: 93.4-99.2 %), PPV was 86.4 % (95 % CI: 76.2–96.5 %), and NPV was 88.6 % (95 % CI: 83.9–93.3 %) compared to the culture respectively (Table 3).

**Table 3:** Sensitivity, specificity, and Predictive values of AFB smear and Xpert MTB/RIF assay using culture as a gold standard

Method and test type	Sensitivity %(95 % CI)	Specificity %(95 % CI)	PPV %(95 % CI)	NPV %(95 % CI)
AFB Smear	36.2 (23.8–48.6)	99 4 (98.2–100)	95.5 (86.8–100)	81.3 (75.9–86.7)
GeneXpert MTB/RIF assay				
Smear negative	48.6(32.5–64.8)	96.9(94.2–99.6)	78.3(61.4–95.1)	89.1(84.5–93.8)
Smear positive	95.2(86–100)	N/A^a^	95.2(86–100)	N/A
Total (SPTB & SNTB)	65.5(53.3–77.7)	96.3 (93.4–992)	86.4(76.2–96.5)	88.6(83.9–93.3)

^a^N/A – Not applicable, SPTB – Smear positive TB, SNTB – Smear negative TB

Xpert MTB/RIF gives a semi-quantitative result (as very low, low, medium, and High) when the test detects MTBC. Eight (18.2 %) samples had a high semi-quantitative result (average cycle threshold (CT) value of 14.3), 10(22.7 %) samples had a medium result (average CT value of 19.1), 12(27.3 %) samples had a low result (average CT value of 27.0), and 14 (31.8 %) samples demonstrated a very low semi-quantitative result (average CT value of 33.1). The average Ct value was significantly lower in smear-positive compared with smear-negative cases (19.9 vs. 28.8; *P* < 0.05). An inverse association between Ct of Xpert MTB/RIF assay and AFB smear grading was found. Time to growth in MGIT medium and of Xpert MTB/RIF result was also compared. The median time required to grow in liquid medium for Xpert positive samples with MTBC isolates was statistically lower than that for Xpert negative samples (9 versus 17 days; *P < 0.05*).

A total of 38 (86.4 %) valid RIF result were obtained from positive Xpert MTB/RIF assay. Among 38 specimens with MTB detected by Xpert, rifampicin resistance was detected in 9.0 % (4/38) of specimens. Indeterminate RIF results were recorded in 13.6 % (6/44) specimens, among these rifampicin indeterminate result, 4 results occurred in smear negative-culture positive samples and 2 results occurred in smear negative-culture negative samples with very late Ct (33–37). Among 4 rifampicin resistance result, 3 strains were identified from smear-negative culture-positive samples, and 1 strain from smear-positive culture-positive sample.

## Discussion

In the present study, high prevalence (26.4 %) of culture confirmed TB was found among the study participants. The conventional diagnostic tool, smear microscopy detected only 9.3 % (21/227) of cases. Xpert MTB/RIF detected 16.7 % (38/227) of cases. Xpert MTB/RIF diagnosed more patients than did smear microscopy in the study.

Overall sensitivity of Xpert MTB/RIF was 65.5 % (95 % CI: 53.3 – 77.7 %). The sensitivity observed in this study was comparable to Dorman SE, et al., who reported 62.6 % in South Africa [9]. Balcha et al. [10] also reported 67.6 % sensitivity in Adama, Ethiopia, which was consistent with the findings of our study. However reports in literature indicate that the sensitivity of Xpert MTB/RIF is as high as 96.7 % in Lima, Peru and 100 % in New-Zealand [11, 12]. Direct comparisons of overall sensitivity may be challenging due to different methodologies and sample type applied for the study.

When the specimen stratified by smear microscopy result, Xpert MTB/RIF demonstrated 95.2 % (95 % CI: 86.1–100 %) sensitivity for smear-positive, culture-positive and 48.6 % (95 % CI: 32.5–64.8 %) for smear-negative, culture-positive specimen. The results for smear-positive samples do not differ from other studies conducted in different places which have demonstrated a sensitivity ranging from 95.6 to 100 [11, 13, 14]. However, the sensitivity found for smear-negative, culture-positive was lower than reported by others ranging from 61 % to 76.9 % [11, 14–16]. Sensitivity of 48.6 % found in this study is consistent with similar results reported by previous studies published in South Africa by Lawn SD et al. (43.%) [13]. The sensitivity in smear-negative, culture-positive samples also varied substantially in different studies, ranging from 43.4 % to 91.7 % [11, 13–16]. Using culture as a gold standard, 34.4 % (20/58) false negative Xpert results were detected in smear negative specimens. These false negative results in smear negative specimen substantially decreased the sensitivity of Xpert MTB/RIF assay in this study. The possible explanation for this discrepancy could be differences in study designs and the presence of PCR inhibitors or insufficient nucleic acid material in the specimens. Despite its low sensitivity in smear negative specimen, Xpert MTB/RIF had almost 31 % increase in TB case detection compared with smear microscopy alone. Xpert MTB/RIF detected 18 more cases than smear microscopy did.

Specificity of Xpert MTB/RIF assay found in this study was 96.3 % (95 % CI: 93.4 – 99.2 %). The result of specificity is comparable with other studies conducted in different places which have shown a specificity ranging from 94.1 to 100 [10–17]. However, 6 (2.6 %) patients had positive Xpert MTB/RIF results but no bacterial growth in MGIT or LJ media. All specimen tested positive in the ‘very low and low’ range of Xpert MTB/RIF detection with average Ct of 28.3. Possible reasons might be also non-viable organism excretion as suggested to occur in self- treating patient or in patent receiving anti-TB treatment but not declaring they are on treatment at the beginning of the study [14, 17].

Different studies reported that the semi-quantitative MTB complex load estimates from the Xpert MTB/RIF assay have good correlation with the smear status of the patients. Similarly this study found good correlation between smear grading and Xpert MTB/RIF assay load as demonstrated by other studies [10, 13, 14]. Consistent with previous report with higher Xpert MTB/RIF assay loads were associated with decreased MGIT culture TTP in our study [10]. Low semi-quantitative Xpert MTB/RIF assay loads were significantly more common in smear-negative than smear positive specimen.

To minimize a relative high cost of Xpert MTB/RIF assay, it is important to control the chance of getting failed test reports. Invalid and error reports have important cost implication. A total of 4 invalid Xpert MTB/RIF test results were obtained in this study. These results counted as test failure. We found acceptably low occurrence of test failure rate (1.8 %) in the current study. The frequency of invalid results was lower than previously reported studies (4.5 % to 6.2 %) [10, 12].

## Conclusions

This study provided information about on how Xpert MTB/RIF tests perform at the point of care and where the assay can have greatest impact on patient care in remote health care facility. Although Xpert MTB/RIF assay demonstrated high sensitivity in detecting MTBC in sputum specimens compared with conventional AFB smear microscopy, it demonstrated suboptimal sensitivity in smear negative patients compared to conventional culture. Requirement of minimal training to run the test and simultaneously detection of MTBC and RIF resistance would make Xpert MTB/RIF assay to be a good candidate to roll-out in national TB control programs. However, cost, constant supply of consumables, short expiry dates of cartridge, and requirement of annual instrument calibration will be a major challenge for the roll-out of Xpert MTB/RIF testing in Ethiopia. All the above points should be considered when assessing Xpert MTB/RIF in national TB control programs to use for detection of TB in TB suspect patient. So, further studies are required to clarify operational difficulty, challenges, and limitations in role-out Xpert MTB/RIF in current TB control/treatment algorithms in the country.

## Abbreviations

DST: Drug susceptibility testing
HIV: Human immunodeficiency virus
LJ: Lowenstein Jensen
MDR: Multi-drug resistant
MGIT: Mycobacteria growth indicator tube
MTB: *Mycobacterium tuberculosis*
MTBC: Mycobacterium tuberculosis complex
RIF: Rifampicin
TB: Tuberculosis
WHO: World Health Organization
